# Dendritic cells as Achilles’ heel and Trojan horse during varicella zoster virus infection

**DOI:** 10.3389/fmicb.2015.00417

**Published:** 2015-05-08

**Authors:** Günther Schönrich, Martin J. Raftery

**Affiliations:** Institute of Medical Virology, Helmut-Ruska-Haus, Charité-Universitätsmedizin Berlin, Berlin, Germany

**Keywords:** dendritic cells, viral pathogenesis, viral immune evasion, varicella zoster virus, herpesviruses

## Abstract

Varicella zoster virus (VZV), a human alphaherpesvirus, causes varicella and subsequently establishes latency within sensory nerve ganglia. Later in life VZV can reactivate to cause herpes zoster. A reduced frequency of VZV-specific T cells is strongly associated with herpes zoster illustrating that these immune cells are central to control latency. Dendritic cells (DCs) are required for the generation of VZV-specific T cells. However, DCs can also be infected *in vitro* and *in vivo* allowing VZV to evade the antiviral immune response. Thus, DCs represent the immune systems’ Achilles heel. Uniquely among the human herpesviruses, VZV infects both DCs and T cells, and exploits both as Trojan horses. During primary infection VZV-infected DCs traffic to the draining lymph nodes and tonsils, where the virus is transferred to T cells. VZV-infected T cells subsequently spread infection throughout the body to give the typical varicella skin rash. The delicate interplay between VZV and DCs and its consequences for viral immune evasion and viral dissemination will be discussed in this article.

## Introduction

In 1943 Helmut Ruska discovered that the virus particles isolated from varicella lesions and those isolated from zoster lesions had identical morphology (Ruska, 1943). The virus was subsequently named varicella zoster virus (VZV). Together with herpes simplex virus (HSV) type 1 and 2 it represents the human alphaherpesviruses (Arvin and Gilden, 2013). After transmission VZV replicates in epithelial cells of the upper respiratory tract. Subsequently, cell-associated viremia is observed which is followed by the appearance of skin infection, giving the typical varicella rash (commonly known as chickenpox) after an incubation period of 10–21 days.

After primary infection has been resolved VZV establishes latency in the dorsal ganglia of nerve cells that innervate the affected skin. It can reactivate, mostly in immunosuppressed individuals and in elderly adults, resulting in herpes zoster (commonly known as shingles) after anterograde transport to the corresponding dermatome. Skin blisters that occur during varicella and zoster harbor high numbers of infectious particles. The main route of transmission is through cell free virus particles that are carried by aerosols of infectious respiratory tract secretions or vesicular fluid. Pathological consequences of VZV include congenital varicella syndrome (Sauerbrei and Wutzler, 2000) and postherpetic neuralgia (PHN; Gnann and Whitley, 2002).

More than 30 years ago a live attenuated vaccine (vOka) was developed from the Oka parental strain (pOka; Takahashi et al., 1974). Intriguingly, vOka is a mixture of pOka genomes with distinct patterns of mutations (Gomi et al., 2002). In 1995 vOka was introduced in the United States and is now licensed for routine use in many countries worldwide. This resulted in significant reduction of the VZV-associated disease burden. Although complications are rare in childhood infection, adult infection, common in tropical countries, induces a more severe clinical course (Lee, 1998). The incidence and severity of herpes zoster increases with age. It was first postulated in 1965 that this is due to a decline in VZV-specific immunity (Hope-Simpson, 1965). It is now accepted that boosting VZV-reactive T cells in older adults using vOka is effective in preventing herpes zoster (Oxman et al., 2005; Gagliardi et al., 2012). Dendritic cells (DCs) are professional antigen-presenting cells that are key to a successful antiviral immune response. Upon sensing a pathogen DCs not only mount an innate response but also specifically instruct the adaptive immune system. This results in the activation of effector mechanisms that are tailor-made for the infecting pathogen (Medzhitov, 2007; Volz et al., 2012; Maisonneuve et al., 2014). Thus, DCs bridge the innate and adaptive arms of the immune system and orchestrate the different immune cells. In this review, we will discuss how VZV exploits DCs for its own purposes.

## Tropism of VZV for DC Subsets

In the absence of inflammation (steady state) two main groups of DCs are observed in the skin and mucosa of the respiratory tract: conventional DCs (cDCs) and Langerhans cells (LCs; Pulendran, 2015). They migrate continuously via the lymphatics to draining lymph nodes. This migration is considerably increased during inflammation. cDCs subsets are derived from committed progenitor DCs in the bone marrow that traffic through the blood to peripheral tissue where they undergo a final differentiation step. In contrast, LCs are maintained independently of circulating progenitor DCs originating in the bone marrow. Migratory DCs form an intricate network that functions as a sentinel system in the peripheral tissue. Upon encounter with pathogens such as VZV they take up antigen and migrate through the lymphatics to the draining lymph nodes. They can localize in the T cell zone to present pathogen-derived antigen and activate specific T lymphocytes. Plasmacytoid DCs (pDCs), which produce large amounts of type I interferon (IFN) in response to viruses, fully differentiate in the bone marrow before they populate the lymphoid organs and become resident DCs.

Under inflammatory conditions during infection human monocytes can also develop *in vivo* into so-called inflammatory DCs (mo-DCs; Segura et al., 2013). Equivalent DCs can be generated *in vitro* by culturing CD14^+^ human monocytes in the presence of granulocyte-macrophage colony-stimulating factor (GM-CSF) and interleukin (IL)-4 and subsequently exposing them to maturation stimuli such as CD40L or tumor necrosis factor (TNF)-α (Sallusto and Lanzavecchia, 1994). These mo-DCs represent the most frequently used *in vitro* DC model and are permissive to VZV (Abendroth et al., 2001b; Morrow et al., 2003; Hu and Cohen, 2005; Gutzeit et al., 2010). VZV-infected immature mo-DCs do not undergo DNA fragmentation (Abendroth et al., 2001b) but can acquire other characteristics of cell death such as impaired membrane integrity and phosphatidylserine exposure (Gutzeit et al., 2010). The open reading frame (ORF) 47 encodes a serine/threonine protein kinase and promotes VZV replication in immature mo-DCs thereby enhancing spread of VZV to other cells (Hu and Cohen, 2005).

In the skin VZV affects both the dermis, where migratory cDCs reside, and the epidermis, where LCs are located (Nikkels et al., 1993). Both LCs and dermal cDCs isolated *ex vivo* are susceptible to VZV infection (Gutzeit et al., 2010). In VZV-infected human skin lesions the frequency and distribution of DC subsets changes most likely due to immigration and emigration similar to HSV-infected mouse skin (Eidsmo et al., 2009). The frequency of LCs is drastically reduced implying activation and migration of LCs to draining lymph nodes (Gutzeit et al., 2010; Huch et al., 2010). In contrast, an influx of other DCs such as inflammatory mo-DCs (Gutzeit et al., 2010) and pDCs (Gerlini et al., 2006; Huch et al., 2010) is observed. Skin biopsies from varicella lesions show occasional cDCs, pDCs, and LCs that express VZV proteins suggestive of VZV infection. Most likely, LC egress is induced by cytokines released during VZV infection. In accordance, inflammatory cytokines such as TNF-α or IL-1β are known to trigger LC migration (Cumberbatch et al., 1997). It is possible that migratory LCs represent the main route for transport of virus and viral antigen to the lymph nodes.

## Role of DCs in VZV Dissemination

Varicella zoster virus-infected patients are extremely contagious. Primary VZV infection is by droplets of infectious saliva or respiratory tract secretions (Suzuki et al., 2004). Intriguingly, intraepithelial DCs in the lung extend cellular processes into the air lumen allowing continuous surveillance of the luminal surface of the alveoli and airways (Jahnsen et al., 2006) similar to gut DCs (Rescigno et al., 2001). Accordingly, these DCs are probably amongst the first cells to interact with VZV. The DC tropism and resultant migration permits VZV to shuttle to the tonsils and the regional lymph nodes. There, VZV-infected DCs establish intimate contact and infect T lymphocytes, as has been demonstrated *in vitro* (Abendroth et al., 2001b).

**FIGURE 1:**
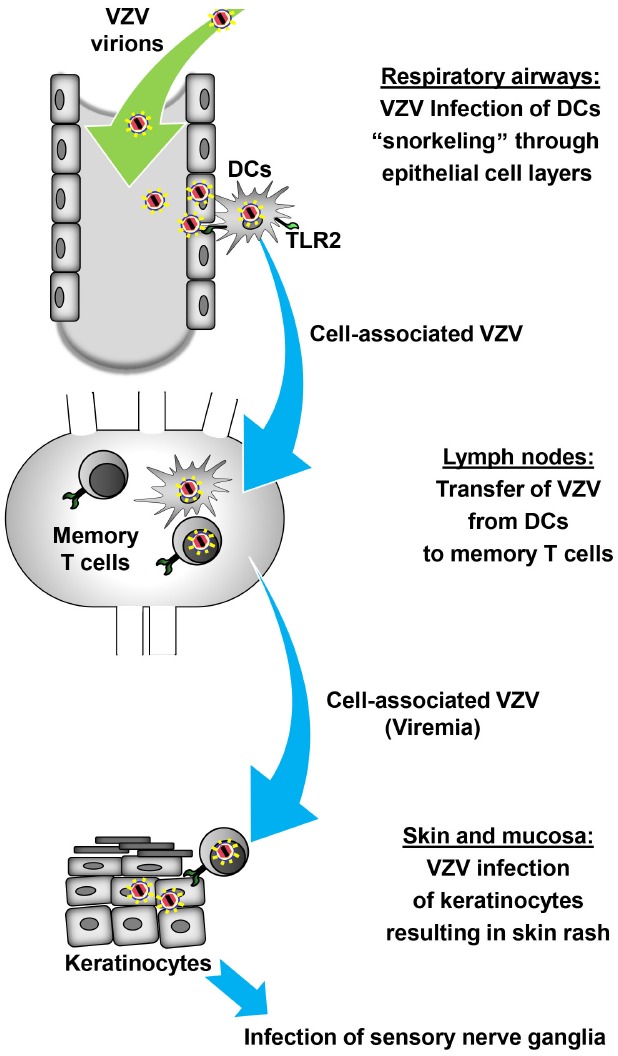
**Model of DC-assisted viral dissemination during primary VZV infection.** After inhalation of VZV-containing aerosols intraepithelial DCs that extend projections into the lumen of the airways, analogous to “snorkeling,” pick up VZV directly or from infected epithelial cells. Upon recognition of VZV through PRRs such as TLR2 intraepithelial DCs and possibly other cells infected with VZV start to migrate to the lung-draining lymph nodes in the first phase of cell-associated viral dissemination. At this site, DCs transfer VZV to memory T cells which subsequently carry VZV in the second phase of cell-associated viral dissemination to the skin. VZV-infected memory T cells infect keratinocytes either directly or indirectly through infection of other skin-resident cells which subsequently transfer the virus to keratinocytes. Finally, VZV replicating in the skin causes the typical rash followed by latent infection of sensory neurons.

Confirming a role for T cells in shuttling virus to the skin VZV lesions appeared in human skin grafts of severe combined immunodeficiency mice (SCID) after injection of VZV-infected human T cells (Ku et al., 2004). Further evidence for the concept of a DC/T cells-axis during VZV dissemination comes from experiments with the closely related simian varicella virus (SVV; Messaoudi et al., 2009). Infected DC-like cells were detected in the lung of African green monkeys after intratracheal infection with recombinant enhanced green fluorescent protein (EGFP)-expressing SVV and transport the virus to the draining lymph nodes where it infects memory T cells (Ouwendijk et al., 2013). Other immune cells in the blood including DCs were also infected with SSV and may contribute to the hematogenous transfer of virus to the skin. The possible DC/T cells-axis during VZV dissemination is illustrated in Figure 1.

There are still open questions concerning this concept. Firstly, it has not been tested whether VZV-infected DCs still respond to chemotactic signals and are able to migrate *in vivo*, although the absence of LC in VZV skin lesions and the ability to infect primary LCs *ex vivo* strongly suggest this (Gutzeit et al., 2010; Huch et al., 2010). Secondly, virus cell-to-cell transmission is more than the sum of virus release and entry as these processes have to be coordinated and channeled to the site of cell-to-cell contact (Mothes et al., 2010). Thus far, the molecular details of how DCs transfer VZV to T cells have not been defined. It is possible but not proven that this process is similar to the DC-to-T cell *trans* infection observed during HIV pathogenesis (Rinaldo, 2013). This *trans* infection requires an intimate DC/T cell contact through membrane molecules and the formation of a virological synapse across which the virions are transferred. In addition, it is unclear which DC subset(s), epidermal and/or dermal DCs, transfers VZV to T cells *in vivo*. Finally, it has not been investigated whether mature DCs are more effective in transferring VZV to T cells as it is the case with HIV (Izquierdo-Useros et al., 2007).

## VZV-associated Innate Signaling

Although DC and T cells can serve as Trojan horses, they also represent an enormous risk to the virus: DCs efficiently initiate the antiviral defense. Innate immune responses are triggered by virus-associated components through pattern recognition receptors (PRRs) such as toll-like receptors (TLRs; Vandevenne et al., 2010). Intact VZV virions activate innate signaling in monocytes and macrophages via TLR2 located on the cell membrane (Wang et al., 2005). However, the corresponding viral ligand has not yet been defined. Possibly, VZV-encoded glycoproteins in the viral envelope are involved similar to HSV (Leoni et al., 2012). A recent publication has shown that the VZV-encoded deoxyuridine triphosphate nucleotidohydrolase (dUTPase), a non-structural viral protein, induces TLR2-dependent secretion of inflammatory cytokines in DCs generated from CD34^+^ human stem cells (Ariza et al., 2014). Similar to other herpesviruses, the VZV-encoded dUTPase may be released by infected cells after induction of apoptosis or pyroptosis before it binds to TLR2 on the cell membrane. Alternatively, VZV-encoded dUTPase could be released through exosomes as demonstrated for Epstein-Barr virus, a gammaherpesvirus (Ariza et al., 2013).

Herpesviruses have developed diverse and numerous strategies to interfere with innate immune responses (Vandevenne et al., 2010). VZV prevents translocation of NF-κB subunits into the nucleus of mo-DCs (Sloan et al., 2012). This inhibition requires the E3 ubiquitin ligase domain of VZV ORF61 and is downstream of triggering receptors such as TLR3, TLR8, and TLR9. It is possible that further VZV proteins target NF-κB signaling. Moreover, it has been demonstrated that VZV ORF61 down-modulates the interferon regulatory factor (IRF) 3-mediated IFN-β pathway (Zhu et al., 2011). In accordance, infection of rhesus macaques with recombinant SVV lacking ORF61 resulted in an increased frequency of pDCs and increased expression of IFN-β as well as IL-6, which is dependent on NF-κB activation (Meyer et al., 2013). SVV ORF61 encodes a 54.1-kDa polypeptide that is homologous to VZV ORF61 and to other alpha herpesvirus proteins that are encoded in similar regions of the viral genome such as HSV-encoded infected cell protein 0 (ICP0). HSV ICP0 also inhibits the induction of IFN-stimulated genes with the help of its E3 ubiquitin ligase domain (Eidson et al., 2002; Lin et al., 2004). Moreover, several other VZV proteins interfere with type I IFN-mediated responses by targeting IRF3 activation including ORF62 (Sen et al., 2010) and ORF47 (Vandevenne et al., 2011). These viral proteins are most likely also functional in VZV-infected pDCs as they no longer produce type I IFN (Huch et al., 2010). In VZV skin accumulations of pDCs were observed (Gerlini et al., 2006; Huch et al., 2010). Similarly, after intrabronchial infection of rhesus macaques with SVV increased frequencies of pDCs are found that correlate with enhanced type I IFN production and a rapid decrease in viral load (Haberthur et al., 2014). These results implicate that pDCs are recruited to the site of VZV replication to support the antiviral host defense, however type I IFN release is inhibited in VZV-infected pDCs by not yet defined mechanisms. Upon stimulation pDCs are also able to present viral antigen and prime virus-specific T cells (Colonna et al., 2004). It is likely but not yet proven that VZV also interferes with T cell stimulation by pDCs.

## Modulation of Adaptive Immune Responses by VZV

T cells not only transport VZV to the skin but also play a pivotal role in controlling VZV during primary infection (Malavige et al., 2008). Decreased VZV specific immunity in elder individuals is associated with increased risk for herpes zoster. This strongly suggests that T cells also control VZV reactivation (Hope-Simpson, 1965; Burke et al., 1982; Levin et al., 2003). At the time being it is unclear whether and how DCs contribute to control of latent VZV by memory T cells.

**FIGURE 2:**
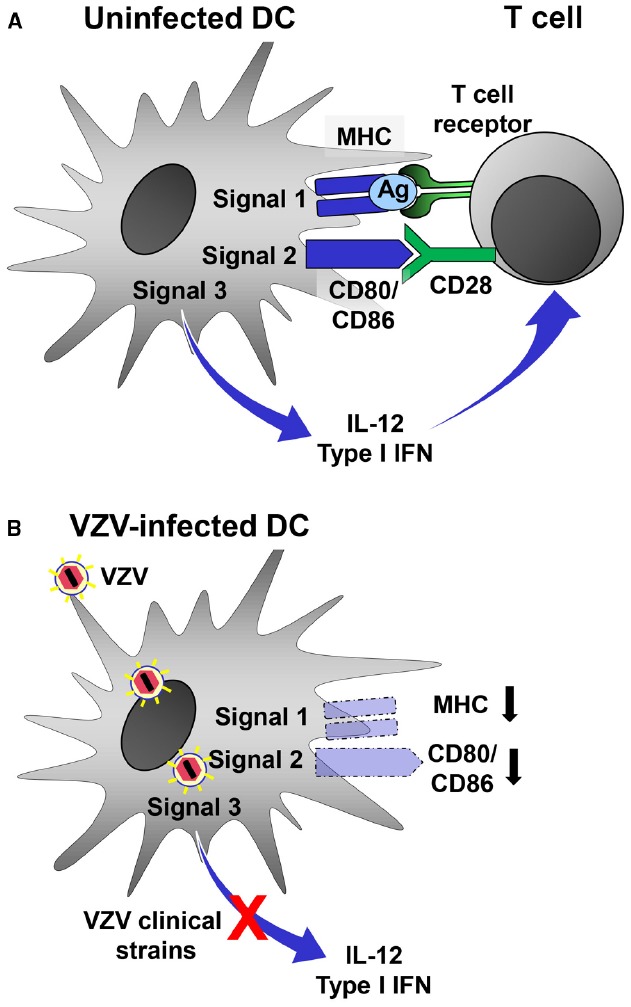
**Evasion of DC function by VZV. (A)** Stimulated uninfected DCs can induce efficient antiviral Th1-like adaptive immune responses by providing three signals to antiviral T cells. Virus-derived antigen (Ag) is presented by MHC molecules (signal 1) and recognized by the T cell-receptor (TCR) on T cells. Costimulatory molecules such as CD80 and CD86 interact with CD28 expressed by T cells (signal 2). DCs integrate stimuli received through a combination of different PRRs and release type I IFN and IL-12. **(B)** VZV-infected DCs are no longer able to deliver signal 1–3 to antiviral T cells. MHC as well as costimulatory molecules are downregulated. Moreover, VZV clinical strains but not the vaccine strain interfere with PRR-induced innate signaling resulting in the inability to release type I IFN and IL-12. Together, these functional changes prevent VZV-infected DCs to mount an effective antiviral Th1-like adaptive immune response.

Varicella zoster virus interferes with T cell responses similar to most other herpesviruses (Smith and Khanna, 2013). For this purpose it targets DCs, which promote Th1-like adaptive immune responses by providing three major signals to T cells (Figure 2A). Signal 1 is delivered by antigen presentation through major histocompatibility complex (MHC) molecules whereas signal 2, also called costimulatory signal, is mediated by CD40, CD80, and CD86. Proinflammatory cytokines released by DCs, mainly IL-12 and type I IFN, represent a third signal that drives Th1-like adaptive immune responses. VZV clearly weakens signal 1–3 by targeting DCs (Figure 2B). After infection of mature mo-DCs VZV impairs signal 1 by downregulation of MHC class I (Abendroth et al., 2001a) as well as signal 2 by reducing surface density of CD80 and CD86 (Morrow et al., 2003). As a consequence of these phenotypic changes VZV-infected mo-DCs have a reduced capacity to stimulate allogeneic T lymphocytes. On the molecular level it has been found that VZV ORF66, a protein kinase, retains mature MCH class I complexes in the *cis*/medial Golgi (Abendroth et al., 2001a; Eisfeld et al., 2007). By which mechanism(s) VZV downregulates costimulatory molecules on mature mo-DCs is unknown. In addition, VZV-infected DCs also express diminished levels of CD83, an important maker of mature DCs, similar to mature DCs infected with HSV, or human cytomegalovirus (Kruse et al., 2000; Morrow et al., 2003; Senechal et al., 2004).

Central to viral immune evasion is the third signal which can be blocked only by VZV clinical strains but not by vOka, the VZV vaccine strain (Gutzeit et al., 2010). Efficient IL-12 secretion and Th1-polarization by DCs requires priming of DCs by synergistic signaling through multiple PRRs (Schulz et al., 2000; Napolitani et al., 2005; Roses et al., 2008) and is facilitated by innate T cells (Leslie et al., 2002; Fujii et al., 2007). In fact, TLRs control key functions of DCs and mediate signals that play a pivotal role in initiation of adaptive immune responses (Iwasaki and Medzhitov, 2004). In accordance, the VZV-associated block of IL-12 secretion was due to impaired signaling downstream of TLR2 (Gutzeit et al., 2010). TLR2 is triggered by VZV, most likely by a viral glycoprotein within the viral envelope and dUTPase released from VZV-infected cells (Wang et al., 2005; Ariza et al., 2013). Peripheral blood mononuclear cells from humans vaccinated with vOka produce high amounts of IL-12 after restimulation *in vitro* (Jenkins et al., 1998). This is in line with the notion that vOka instructs DCs to promote an efficient Th1-type of adaptive immune response. This instructive capability makes vOka a prime candidate vector for the development of novel vaccines. Its genome is large enough to accommodate even large DNA inserts encoding antigenic structures from other viral pathogens using bacterial artificial chromosome technology (Somboonthum et al., 2007; Matsuura et al., 2013).

## Concluding Remarks

In this article we have discussed how VZV, one of the most successful human pathogens, exploits DCs to disseminate in the human organism, evade the antiviral immune responses and cause disease. In contrast, vOka, the only live attenuated vaccine available against human herpesviruses, does not cause significant disease. Although both wild type VZV and vOka target DCs only DCs infected with vOka can be instructed to become a Th1-promoting antigen-presenting cell. It will be important in the future to precisely define on the molecular level how DCs infected with wild type VZV differ from those infected with vOka. Moreover, it is necessary to pinpoint the viral proteins responsible. A better understanding of these differences could allow to design desperately needed vaccines against other human herpesviruses.

### Conflict of Interest Statement

The authors declare that the research was conducted in the absence of any commercial or financial relationships that could be construed as a potential conflict of interest.
